# Breeding pairs with color aberrations in oriental reed warblers

**DOI:** 10.1002/ece3.11676

**Published:** 2024-07-03

**Authors:** Laikun Ma, Peng Pan, Wei Liu, Jianhua Hou, Wei Liang

**Affiliations:** ^1^ School of Life Sciences Hebei University Baoding China; ^2^ Department of Biology and Food Science Hebei Normal University for Nationalities Chengde China; ^3^ Ministry of Education Key Laboratory for Ecology of Tropical Islands, Key Laboratory of Tropical Animal and Plant Ecology of Hainan Province, College of Life Sciences Hainan Normal University Haikou China

**Keywords:** albinism, color aberration, egg recognition ability, leucism, oriental reed warbler

## Abstract

In 2017, one pair of Oriental reed warblers (*Acrocephalus orientalis*) with color aberrations was found in Yongnianwa National Wetland Park, Hebei, China. The female bird exhibited white feathers on the head, neck, and upper back, and the base of the beak was flesh‐red in color. The male had a few feathers on the outer edges of the left and right primary wing coverts that were white, which was determined to be leucism after analysis. The breeding pairs laid their first egg on May 29, with a clutch size of four eggs. After an incubation period of 13 days, two chicks hatched on June 13, 2017. The nest was found empty on June 20 when the chicks were 7 days old and before fledging age; therefore, it was presumed that the chicks had been predated. A white parrot egg was added to the nest during the incubation period to test the egg recognition ability of breeding pairs and was successfully rejected. To the best of our knowledge, this is the first report of color aberrations in the Oriental reed warbler, and we found that this color aberrations did not affect some reproductive and antiparasitic behaviors of the birds, but whether it affects their breeding success needs to be further studied.

## Graphical abstract

One pair of Oriental reed warblers (*Acrocephalus orientalis*) with color aberrations was found in Yongnianwa National Wetland Park, Hebei, China. This is the first report of color aberrations in the Oriental reed warbler. Color aberrations this color aberrations did not affect some reproductive and antiparasitic behaviors of the birds.

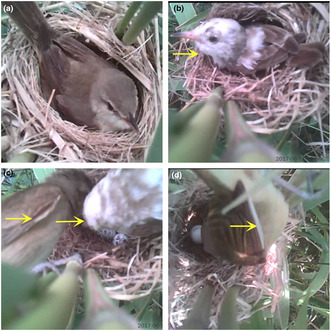

## 1 | INTRODUCTION

Biochromes, structural colors, and a combination of both can contribute to the colorful plumage of birds. Melanin is the most common biochrome is divided into eumelanin and pheomelanin variants. Melanin is produced by melanocyte cells that are widely found in the skin, hair, feather follicles, and eyes (Dupin & Le Douarin, 2003; van Grouw, 2013). Due to factors such as disease, poor nutrition, or genetic variation, differences in melanin synthesis and distribution can result in color aberrations divergent from normal coloration. Among such cases, color aberrations caused by non‐genetic factors, such as adverse external environments, can gradually return to normal after the external conditions improve (van Grouw, 2006, 2013).

The phenomenon of abnormal body color caused by color aberrations in birds has long been of interest to ecologists, ornithologists, birdwatchers, and nature lovers. It can be classified into different types of color aberrations based on different pathogenic factors and phenotypic characteristics, with the most common being albinism, leucism, and progressive graying (Mahabal et al., 2016; van Grouw, 2013). Albinism is a genetic mutation that leads to the lack of the tyrosinase necessary for melanogenesis in the body, manifesting as a pathological condition in which the feathers, skin, and eyes, can become colorless. Leucism is a genetic or congenital failure of melanoblasts to successfully migrate to the surface of the skin, resulting in a defect in the development of melanin‐producing pigment cells in all or part of the body. In birds, leucism presents as the loss of melanin in growing feathers, resulting in white feathers or skin in all or part of the body. Progressive graying is the gradual loss of melanin‐synthesizing pigment cells in all or some areas of the body after an organism reaches a specific age, as evidenced in birds by the whitening of feathers with molting and aging. With the advancement of bird‐watching equipment, the expansion of bird‐watching groups, and the development and application of the internet, more and more birds with color aberrations are being discovered and reported (Husby, 2017; Mahabal et al., 2016; Ray, 2021; Tinajero & Rodríguez‐Estrella, 2010; Zbyryt et al., 2021). However, there are many misuses of avian color aberration types (van Grouw, 2006; Zbyryt et al., 2021) and there are only a few cases of in‐depth studies of the color aberration that occur in birds (e.g. Camacho et al., 2022; Izquierdo et al., 2018), especially in terms of how it affects behavior or reproduction (but see Ellegren et al., 1997; Bensch et al., 2000; Colombo et al., 2018).

The Oriental reed warbler (*Acrocephalus orientalis*) belongs to the genus *Acrocephalus* in the family Acrocephalidae (Passeriformes) (Zheng, 2023) and is a common breeding bird in reed wetlands with abundant populations. It is one of the main hosts of the common cuckoo (*Cuculus canorus*) in the plains, and the two have reached an advanced stage of coevolution (Li et al., 2016; Ma et al., 2022). In this study, one pair of Oriental reed warblers with color aberrations was found during fieldwork, and their reproductive process and egg recognition ability were studied.

## 2 MATERIALS AND METHODS

### 2.1 Study area and species

The study area is located in Yongnianwa National Wetland Park (36°40'–41' N, 114°41'–45' E) in Hebei Province, China. Yongnianwa is a natural depression with an elevation of only 40.3 m. It has a well‐developed water system with numerous tributaries and water accumulation year‐round. It is located in a warm, temperate, semi‐humid continental monsoon climate zone with four distinct seasons and simultaneous rainfall and heat. The average annual rainfall is 527.8 mm, mostly in the summer, and the average annual temperature is 12.9°C. The main vegetation type of the wetland is reed (*Phragmites australis*) that is interspersed with cattail (*Typha latifolia*) and other herbs (Ma et al., 2018). The common cuckoo is the most commonly seen brood parasite in this area, and the Oriental reed warbler is one of the main hosts of the common cuckoo in reed wetlands with a cuckoo parasitism rate of about 14.8% (Ma et al., 2018, 2022).

### 2.2 Field experiments

**FIGURE 1:**
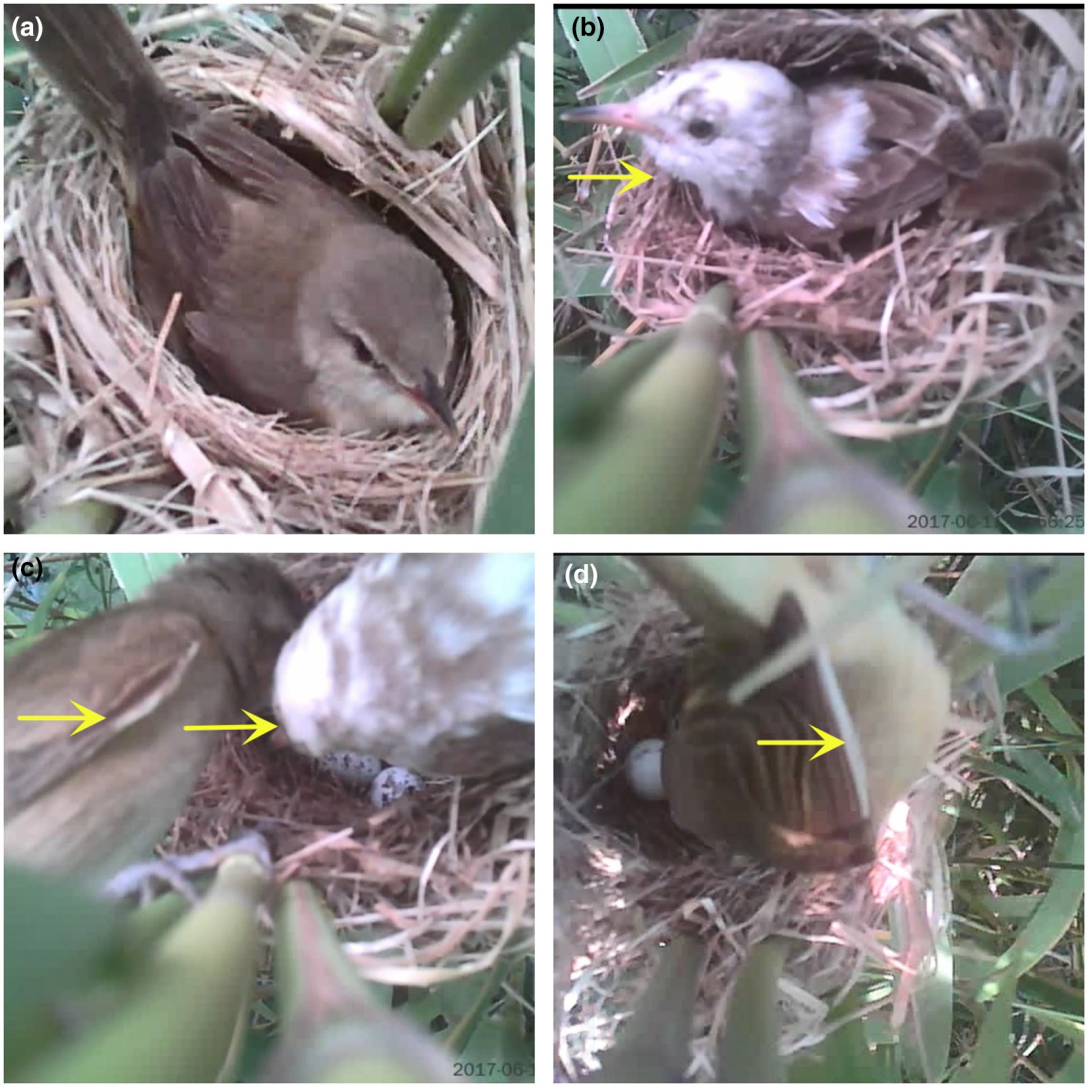
Female and male Oriental reed warbler showing color aberration for leucism. (a) refers to normal plumage; (b) refers to leucistic female with white feather in head, neck and upper back; (c) refers to leucistic male with white feather in right wing; (d) refers to the same leucistic male with white feather in left wing.

Fieldwork was conducted during the 2017 breeding season and one pair of the Oriental reed warbler with color aberrations in the nesting stage was found during a systematic search for breeding nests (Figure 1). Their breeding process was continuously monitored and tested for egg recognition as they were in the incubation period, and the process was recorded with a mini‐camera. Previous studies showed that replacement or direct addition of one experimental egg does not affect host egg recognition (Davies & Del Brooke, 1988), so one white model egg was added directly to the breeding nest to observe the parents' response over 6 days (Moksnes et al., 1991). If the host did not reject the model egg within 6 days, it was considered accepted; if the model egg disappeared or the parent bird abandoned the nest within 6 days, it was considered rejected.

## 3 | RESULTS

Compared to the normal breeding pairs of the Oriental reed warbler (Video S1), in this breeding pair, the head, neck, and upper back feathers of the female Oriental reed warbler were whitened and the base of the beak had flesh‐red coloration. The outer edge of a few feathers of the left primary and right primary feathers on the male were whitened, while the eyes of both birds were normal in color (Figure 1; Video S2). The nest was found empty on June 20 when the produced chicks were 7 days old. As the chicks had not reached the fledging age, it was presumed to have been predated and that the breeding failed. The white model egg added during the incubation period was successfully rejected by the parents.

## 4 | DISCUSSION

Albinism, leucism, and progressive graying are common and frequently misinterpreted types of color aberrations in birds, with leucism and progressive graying often misdescribed as partial albinism (Bensch et al., 2000; van Grouw, 2006; Zbyryt et al., 2021). Albinism is caused by a mutation that disrupts the pathway of melanin synthesis that manifests as the absence of melanin throughout the body and therefore, does not appear as partial albinism. Furthermore, albino birds have red eyes, which is the biggest difference from leucism and progressive graying. Leucism and progressive graying are phenotypically similar and more difficult to distinguish in the field. In the Leucism, the areas of color aberrations are mostly patchy or bilaterally symmetrical and whitening is more commonly observed on the head, abdomen, and wingtips, although it may also appear on the legs and beak. The pathological features can appear in juveniles and do not change with age. In contrast, progressive graying occurs in birds that reach a certain age and the area of aberrations is mostly random and gradually expands as the bird ages and molts until the whole body turns white. The legs and beak are mostly unaffected in the early stages of progressive graying (van Grouw, 2012). In this study, the female Oriental reed warbler showed white plumage patches on the head and neck which in low proportion and was more symmetrical. In addition, the flesh‐red color on the base of the beak was similar to that of newborn chicks, suggesting color aberrations due to leucism. The male bird displayed aberrations in a more symmetrical pattern on both flight feathers, suggesting it was due to leucism. Small amounts of whitening have been observed in its sister species, the great reed warbler (*Acrocephalus arundinaceus*), which can present whitening on its flight feathers (Bensch et al., 2000), similar to the male observed in this study.

In most birds, plumage color may be an important factor for sexual selection (Hill, 2006), and color aberrations may affect the success of pairing (Truax & Siegel, 1982). Additionally, birds with color aberrations, especially albinism, are more conspicuous in the population and prone to predation, resulting in a lower survival rate (Slagsvold et al., 1988; Izquierdo et al., 2018; but see Camacho et al., 2022; Stephenson et al., 2022). In this study, both the male and female Oriental reed warblers were leucistic individuals with color aberrations, and no other individuals with color aberrations were found in the local population with a rate of 0.73% according to number of breeding nests (Ma et al., 2021). This suggests that their successful pairing may be the result of adaptive selection according to similar plumage phenotype. Previous studies on Japanese quails (*Coturnix japonica*) have also found that plumage phenotype affects their mating preferences (Blohowiak & Siegel, 1983; Truax & Siegel, 1982). The breeding pair had similar breeding characteristics to normal birds in terms of clutch size and incubation period. They also presented similar egg recognition and rejection abilities adapted to the parasitic cuckoo as other Oriental reed warblers, suggesting that their color aberrations did not affect their partial breeding or antiparasitic behaviors (e.g., egg recognition ability). However, the chick of this breeding pair was predated during the brooding period, resulting in reproductive failure. This is inconsistent with previous reports of breeding success involving individuals with color aberrations (Bensch et al., 2000; Colombo et al., 2018) and might be due to the high predation rates inherent in the region (Ma et al., 2021). Additionally, as both male and female parents were leucistic individuals, as the age of the chick increased during the brooding period, the parents were frequently near the nest; since the white feathers were more conspicuous in the environment, this may have increased the probability of the chick being found by predators. However, the effect of color aberrations on bird reproduction should be studied in more depth.

In conclusion, we found that the partial reproductive characteristics of leucistic Oriental reed warblers, such as clutch size and incubation period, were similar to those of normal individuals, and that they were able to recognize foreign eggs. However, more extensive investigation and research is needed to monitor color aberrations in this population and other geographic populations, and whether they affect the reproductive success of the population.

## AUTHOR CONTRIBUTIONS

**Laikun Ma:** Formal analysis (equal); investigation (equal); writing – original draft (equal). **Peng Pan:** Investigation (equal); software (equal); visualization (equal). **Wei Liu:** Investigation (equal); software (equal); visualization (equal). **Jianhua Hou:** Conceptualization (equal); resources (equal); supervision (equal); writing – review and editing (equal). **Wei Liang:** Conceptualization (equal); supervision (equal); validation (equal); writing – review and editing (equal).

## FUNDING INFORMATION

This work was supported by the National Natural Science Foundation of China (Nos. 32101242 to LM, and 32270526 to WL).

## CONFLICT OF INTEREST STATEMENT

The authors declare that they have no competing interests.

## Supporting information


Video S1

Video S2

## Data Availability

The data that supports the findings of this study are provided as (Videos S1 and S2) and are available at Dryad (doi_10_5061_dryad_2jm63xsxs_v20240505). Reviewer URL: https://datadryad.org/stash/share/pH82s6oT7yiXTEi7grPSMQvKgVdywZtrSx_B_hUjTRQ.
